# Poor appetite and overeating reported by adults in Australia during the coronavirus-19 disease pandemic: a population-based study

**DOI:** 10.1017/S1368980020003833

**Published:** 2020-09-25

**Authors:** AJ Owen, T Tran, K Hammarberg, M Kirkman, JRW Fisher

**Affiliations:** 1 Centre of Cardiovascular Research and Education in Therapeutics, School of Public Health & Preventive Medicine, 553 St Kilda Rd, Melbourne, Victoria 3004, Australia; 2 Global and Women’s Health, School of Public Health and Preventive Medicine, Monash University, Melbourne, Victoria, Australia

**Keywords:** COVID-19, Overeating, Appetite, Pandemic, Depression

## Abstract

**Objective:**

As a result of the coronavirus-19 disease (COVID-19) pandemic, Australia adopted emergency measures on 22 March 2020. This study reports the effect of the COVID-19 lockdown on appetite and overeating in Australian adults during the first month of emergency measures.

**Design:**

This study reports analysis of data from the population-based, self-completed survey. The main outcome measure was an item from the Patient Health Questionnaire 9 asking: ‘Over the past 2 weeks, how often have you been bothered by poor appetite or overeating?’. Data on sociodemographic factors, symptoms of anxiety and depression, and the impact of COVID-19 and lockdown were also collected. Multivariable logistic regression was used to examine associations with poor appetite or overeating.

**Setting:**

An anonymous online survey available from 3 April to 2 May 2020.

**Participants:**

A total of 13 829 Australian residents aged 18 years or over.

**Results:**

The weighted prevalence of being bothered by poor appetite or overeating in the past 2 weeks was 53·6 %, with 11·6 % (95 % CI 10·6, 12·6) of the cohort reporting poor appetite or overeating nearly every day. High levels of anxiety, concern about contracting COVID-19, being in lockdown with children and reporting a severe impact of the lockdown were associated with increased odds of poor appetite or overeating.

**Conclusions:**

Given the widespread prevalence of being bothered by poor appetite or overeating, universal public health interventions to address emotion-focused or situational eating during periods of lockdown may be appropriate.

The rapidly escalating crisis associated with the coronavirus-19 disease (COVID-19) pandemic arising from severe acute respiratory syndrome coronavirus-2 infection led to a declaration of a state of emergency in Australia on 22 March 2020. All non-essential activity was halted, and people were required by law to stay at home, with the exception of shopping for food and essential items, medical care, work (if unable to work remotely) and limited daily outdoor exercise. Physical distancing measures were required outside the home, and visiting family and friends was prohibited (except for providing essential care).

By the start of May 2020, Australia had experienced a limited spread of the virus causing COVID-19, with fewer than 7000 cases in total (280 cases per million population)^(1)^. The lockdown has had profound social and personal effects, including widespread unemployment and its financial ramifications, disruption to social interaction and concern about contracting a potentially fatal new disease. Evidence of the profound impact of COVID-19 and its associated restrictions on the mental health of people around the world is emerging^(2,3)^, but there is considerable variation in the approaches being taken to manage COVID-19 in countries with different World Bank Income Classifications and transmission levels^(4)^.

Diet is a key driver of health^(5)^ and well-being^(6,7)^, not only through the provision of nutrients which support health (including immune health)^(8)^ or patterns of consumption which influence disease risk^(5)^, but also the social health benefits of shared meals^(9)^. In other countries, the pandemic and associated containment strategies are thought to have affected eating behaviour. In Italy, which experienced a severe outbreak early in the pandemic, one survey found that around 53 % of respondents reported eating more during lockdown and 19·5 % reported weight gain^(10)^. Another survey of adolescents and adults in Italy found that over half reported appetite changes, with some evidence that this varied by age, gender and work status^(11)^. In a survey of adults in Poland, more than 40 % reported eating more and 42 % reported weight change (loss or gain)^(12)^. In China (including sampling in Hubei province, which is thought to be the origin of SARS-CoV-2), dietary diversity was found to be lower in more affected regions^(13)^. Aside from the society-wide impact of confinement on diet, loss of the sense of taste and smell is a common side effect in those who contract COVID-19^(14)^; this may also affect dietary intake.

The objective of this study was to identify self-reports of being bothered by poor appetite or overeating in adults in Australia during the COVID-19 lockdown and learn whether this differs by age, gender or personal and living circumstances. The goal was to contribute to more effective targeting of interventions to improve nutrition and health for those at-risk.

## Methods

This paper reports results of a short, anonymously completed, self-report survey of people living in Australia and aged at least 18 years, with data collected from 3 April 2020 to 2 May 2020. The questionnaire included sociodemographic questions, fixed-response-option questions about the experiences of living with the COVID-19 pandemic and resulting restrictions, and two widely used standardised psychometric instruments of symptoms of depression and anxiety. The survey methods have been published^(2)^.

Symptoms of anxiety were assessed using the Generalised Anxiety Disorder Scale-7^(15)^. The Generalised Anxiety Disorder Scale-7 is a seven-item validated scale assessing frequency of experiencing common symptoms of anxiety in the preceding 2 weeks. Scores of 5–9 represent mild anxiety, 10–14 moderate anxiety and 15–21 severe anxiety. Clinically significant anxiety is defined as a Generalised Anxiety Disorder Scale-7 score ≥ 10.

Symptoms of depression were assessed using the Patient Health Questionnaire 9^(16)^, a nine-item validated scale assessing frequency of experiencing common symptoms of depression in the preceding 2 weeks. Aggregated responses to the nine items provide an indicative scale of depression symptom severity. Patient Health Questionnaire 9 scores of 5–9 represent mild, 10–14 moderate, 15–19 moderately severe and ≥20 severe depressive symptoms. Clinically significant symptoms of depression were defined as Patient Health Questionnaire 9 scores ≥10.

*Bothersome poor appetite or overeating* was determined from item 5 of the Patient Health Questionnaire 9^(16)^, which asks respondents ‘Over the past 2 weeks, how often have you been bothered by poor appetite or overeating?’ with response options of ‘not at all’, ‘several days’, ‘more than half the days’ and ‘nearly every day’.

### Sociodemographic characteristics

The survey collected information on age, gender, postcode, whether born in Australia, living circumstances (who do you live with?) and occupation. Occupation was collected using ten response items (see Ref. (2)); for the purpose of this analysis, they have been collapsed into four groups: ‘Continuing paid work or study from home’, ‘Work or study has been stopped by COVID-19’, ‘Leaving the home to go to work’ and ‘Unpaid caring, welfare benefits, or fixed-income’. This last group included those who reported that they were unpaid carers of children or dependent relatives, retired, had government benefits as their main source of income support or were unemployed before the COVID-19 pandemic. To obtain area-level socio-economic status, the Australian Bureau of Statistics Socio-economic Indices for Areas^(17)^ were derived from a respondent’s postcode, from which quintiles were developed.

Personal impact of COVID-19 restrictions was assessed by the question ‘How badly have the COVID-19 restrictions affected your daily life?’ and rated on a scale of 0–10 (‘not at all’ to ‘very badly’). A high adverse impact of restrictions was defined as a score ≥ 8. Anxiety relating to contracting COVID-19 was assessed by the question ‘How worried are you that you will catch COVID-19?’ and rated on a scale of 0–10 (from ‘not at all worried’ to ‘extremely worried’). Being highly worried about contracting COVID-19 was defined as a score ≥ 8. Personal experience of COVID-19 (being treated or tested for COVID-19, or knowing someone who has contracted COVID-19) was also assessed.

### Statistical analysis

Population prevalence and 95 % CI of people bothered by poor appetite or overeating was estimated, adjusting for differences in sociodemographic characteristics between the sample and the Australian population^(17)^. The adjustment was made using post-stratification weights^(2)^.

Multivariable-adjusted logistic regression modelling of factors associated with being bothered by poor appetite or overeating in the previous 2 weeks was undertaken. The factors included in the model were sociodemographic characteristics, living circumstances, mental health status and experiences of COVID-19 and associated restrictions.

Data analyses were conducted using STATA version 16 (StataCorp.) with only complete data included.

## Results

A total of 13 829 adults (mean age 50·4 (sd 14·9) years) contributed complete data. Their demographic characteristics are presented in Table 1. The weighted prevalence of being bothered at all by poor appetite or overeating in the previous 2 weeks was 53·6 %, with 11·6 % (95 % CI 10·6, 12·6) of respondents bothered by poor appetite or overeating nearly every day (Table 2).

**Table 1:** Sociodemographic characteristics of the study sample

	*n*	%
Total respondents	13 829	
Gender		
Female	10 434	75·5
Male	3328	24·1
Other	67	0·5
Age group in years		
18–29	1337	9·7
30–39	2294	16·6
40–49	2854	20·6
50–59	3064	22·2
60–69	2833	20·5
70+	1447	10·5
Any experience of COVID-19 (whether the respondent had been diagnosed with or tested for COVID-19, or lived with or knew someone with COVID-19)	2147	15·5
Highly worried about contracting COVID-19 (scale score ≥ 8)		
Median	13·9	
Range	13·1–14·8	
High adverse impact of restrictions (scale score ≥ 8)		
Median	25·2	
Range	23·8–26·8	
State		
New South Wales	2753	19·9
Australian Capital Territory	465	3·4
Victoria	6105	44·1
Queensland	1939	14·0
South Australia	836	6·0
Western Australia	1177	8·5
Tasmania	445	3·2
Northern Territory	109	0·8
Living in a major city	9045	65·4
SEIFA quintiles		
Quintile 1 (lowest socio-economic area)	1093	7·9
Quintile 2	1541	11·1
Quintile 3	2228	16·1
Quintile 4	3038	22·0
Quintile 5 (highest socio-economic area)	5929	42·9
Living situation		
With only your partner	4203	30·4
On your own	2660	19·2
With your partner and children	3875	28·0
With children and without a partner	578	4·2
With adult family members	1552	11·2
In a shared house with non-family members	616	4·5
Other	345	2·5
Born overseas	3150	22·8
Occupation		
Continuing paid work or study from home	6767	49·6
Work or study has been stopped by COVID	887	6·5
Leaving the house to work	2262	16·6
Unpaid caring, welfare benefits, fixed-income	3730	27·3

COVID-19, coronavirus-19 disease; SEIFA, Socio-economic Indices for Areas.

Values given as *n* (%), except for ‘Highly worried about contracting COVID-19’ and ‘High adverse impact of restrictions’ which are given as prevalence (%) weighted for State, SEIFA decile, gender and age.

**Table 2:** Prevalence of poor appetite or overeating during coronavirus-19 disease (COVID-19) lockdown in Australia

Over the past 2 weeks, how often have you been bothered by poor appetite or overeating?	Prevalence (%) with weighted data	95 % CI
Not at all	46·4	44·9, 48·0
Several days	25·8	24·5, 27·2
More than half of the days	16·2	15·0, 17·5
Nearly every day	11·6	10·6, 12·6

Prevalence (%) weighted by State, Socio-economic Indices for Areas decile (socio-economic area) gender and age.

Multiple logistic regression analysis indicated that age, gender, socio-economic status, living circumstances, being born overseas, concern about contracting COVID-19 and being highly affected by the COVID-19 pandemic restrictions were associated with odds of being bothered by poor appetite or overeating (Table 3). Above the age of 40 years, after multivariate adjustment, increasing age was incrementally associated with decreasing odds of being bothered by poor appetite or overeating in the previous fortnight.

**Table 3:** Multivariable-adjusted logistic regression model of factors associated with being bothered by poor appetite or overeating in the past 2 weeks

	OR	*P*-value	95 % CI	
Gender				
Female	Ref.			
Male	0·67	<0·001	0·61	0·74
Other	0·45	0·016	0·23	0·86
Age group (years)				
18–29	Ref.			
30–39	0·92	0·349	0·77	1·10
40–49	0·76	0·003	0·64	0·91
50–59	0·64	<0·001	0·54	0·76
60–69	0·49	<0·001	0·41	0·58
70+	0·34	<0·001	0·27	0·42
Moderate, moderately severe or severe (clinically significant) depressive symptoms, PHQ-9 score ≥ 10)	10·58	<0·001	9·12	12·27
Moderate, or severe anxiety (clinically significant) symptoms of anxiety, GAD-7 score ≥ 10)	1·88	<0·001	1·62	2·18
Any experience of COVID-19 (whether the respondent had been diagnosed with or tested for COVID-19, or lived with or knew someone with COVID-19)	1·07	0·244	0·96	1·19
Highly worried about contracting COVID-19 (scale score ≥ 8)	1·27	<0·001	1·13	1·42
High adverse impact of restrictions (scale score ≥ 8)	1·30	<0·001	1·18	1·44
State				
New South Wales	Ref.			
Australian Capital Territory	1·03	0·792	0·82	1·30
Victoria	1·00	0·998	0·90	1·11
Queensland	1·09	0·239	0·95	1·25
South Australia	1·19	0·057	0·99	1·43
Western Australia	1·03	0·698	0·88	1·21
Tasmania	1·10	0·433	0·86	1·41
Northern Territory	0·99	0·960	0·64	1·53
Major city	1·16	0·007	1·04	1·29
SEIFA quintiles				
Quintile 1 (Lowest socio-economic area)	Ref.			
Quintile 2	1·00	0·969	0·83	1·20
Quintile 3	0·96	0·626	0·81	1·14
Quintile 4	0·86	0·080	0·72	1·02
Quintile 5 (Highest socio-economic area)	0·78	0·004	0·65	0·92
Living situation				
With only your partner	Ref.			
On your own	1·38	<0·001	1·23	1·54
With your partner and children	1·19	0·003	1·06	1·33
With children and without a partner	1·48	<0·001	1·20	1·83
With adult family members	1·11	0·166	0·96	1·28
In a shared house with non-family members	1·62	<0·001	1·30	2·02
Other	1·12	0·399	0·86	1·46
Born overseas	0·80	<0·001	0·73	0·88
Occupation				
Continuing paid work or study from home	Ref.			
Work or study stopped by COVID-19	1·01	0·900	0·85	1·20
Leaving the house to work	0·97	0·583	0·87	1·08
Unpaid caring, welfare benefits, fixed-income	0·82	0·001	0·73	0·92

COVID-19, coronavirus-19 disease; GAD-7, Generalised Anxiety Disorder Scale; SEIFA, Socio-economic Indices for Areas; Ref., reference category.

For each variable examined in the model, the given OR are adjusted for all remaining covariates: age, gender, SEIFA, state, residential area classification (major city *v*. outside major cities), being born overseas, living circumstance, occupation, anxiety, depression, concern about contracting COVID-19, felt impact of restrictions, personal experience of COVID-19.

Being male was associated with lower odds of being bothered by poor appetite or overeating, as was high socio-economic advantage (Table 3). Those living alone, with children and without a partner, or in a shared house with non-family members had significantly greater odds of being bothered by poor appetite or overeating than people living in a household with a partner and no children (Table 3).

Being in unpaid caring roles, unemployed before the COVID pandemic and still unemployed, retired or on government welfare benefits were associated with slightly lowered odds of being bothered by poor appetite or overeating compared with those who were continuing paid work or study from home.

### COVID-19 impact, well-being and being bothered by poor appetite or overeating

After adjustment for sociodemographic factors, anxiety and depression, respondents who were highly concerned about contracting COVID-19 and those who felt a high level of adverse impact of the pandemic restrictions had significantly increased odds of being bothered by poor appetite or overeating (Table 3). There was only a small number of respondents who had contracted COVID-19 (*n* 56), which limits the ability to compare this group to the wider sample.

Clinically significant anxiety and depression scores (≥ 10) were each associated with increased odds of being bothered by poor appetite or overeating (Table 3).

## Discussion

These findings demonstrate that, in the first month of COVID-19 pandemic restrictions, more than half of the sampled adults reported that they were bothered by poorer appetite or eating more than usual. Women, those living alone, those living with children and without a partner, those very worried about contacting COVID-19 and those who felt a greater adverse effect of pandemic restrictions, were particularly likely to report being bothered by poor appetite or overeating. Older age, living with adult family members without children, higher socio-economic status, not being in paid work or study before COVID-19 and living in a regional area appeared to reduce the risk of being bothered by appetite and overeating during the pandemic. Clinically significant depression and anxiety were also associated with substantially increased odds of reporting being bothered by poor appetite or overeating.

Emotional eating (eating in response to negative emotions) is a well-described phenomenon^(18)^ and has been implicated in the pathway between depression and obesity^(19)^. Emotional eating is most commonly reported in response to boredom, loneliness and anxiety^(20)^. Stress can suppress appetite^(21)^, and loss of appetite is a common feature of depression and anxiety^(22)^.

Living alone, living with children or living in shared accommodation with non-family members were all associated with increased odds of being bothered by poor appetite or overeating, compared with living with a partner. Eating alone is a known risk factor for malnutrition in older adults^(23)^, and eating either alone or with non-family members is associated with increased risk of obesity when compared with eating with family^(24)^. At the time of the survey, schools were shut to all but the children of essential workers, and most households with children, were ‘learning from home’. While those with sole responsibility for children were most bothered by poor appetite or overeating, those living with a partner and children were also more likely to be bothered by poor appetite or overeating than those living with a partner only. This suggests the possibilities that having children at home during the lockdown may induce stress which affects appetite or overeating or that catering to the dietary needs of children in the home may present additional eating opportunities. Living outside major metropolitan cities was associated with lower odds of being bothered by poor appetite or overeating. Possible explanations for this regional resilience may include feeling somewhat removed from the epicentre of the pandemic (which was concentrated at the time in major cities^(1)^), or perhaps having working and daily lives that were less disrupted by lockdown.

There were important age and gender differences in the reports of being bothered by poor appetite or overeating. Women were more likely to report being bothered by poor appetite or overeating than men. This is consistent with evidence that women tend to report greater weight/body shape concerns, eating concerns and binge eating than men^(25)^. Increasing age was associated with lower odds of being bothered by poor appetite or overeating, despite older age being associated with more worry about contracting COVID-19^(2)^. Possible explanations for this age-related resilience to eating behaviour change may be more habitually stable eating patterns^(26)^, age-related decline in concern about body shape or weight change^(27)^, being more financially secure or having greater life experience of difficult situations and thus more capacity to regulate emotions in the face of the life changes imposed by lockdown. In young adult men, higher anxiety has been reported to be associated with uncontrolled eating, but uncontrolled eating also decreased with age^(22)^. However, a similar pattern of age-related decline in disordered eating was not noted in young women^(22)^.

Environmental factors are known to be associated with food consumption. Overeating is related to the presence of palatable food in the environment^(28)^; with a pandemic-related lockdown, some people may have found themselves working from home in close proximity to the kitchen and its temptations. Those who were not working or studying outside the home before lockdown (and perhaps more accustomed to spending time at home) appeared to fare better in not being bothered by dietary behaviour, even though this group included people on low or fixed incomes (e.g. pensions), a population group generally considered to be at increased dietary risk^(29)^. In a US study of economically disadvantaged women, resistance to emotional eating and favourable social eating behaviours were associated with better diet quality and lower BMI, with evidence that these factors were key to mediating the relationship between food insecurity and BMI in women living on low incomes^(30)^. A feature of the early stages of the first pandemic wave lockdown in Australia was panic-buying or stockpiling of long-life foods such as flour, sugar, dried pasta, rice, biscuits, and bottled and canned foods. Baking and home cooking (notably ‘iso-baking’) were also prominent during this period^(31)^ and may have contributed to an increase in the available food in the home environment.

There appeared to be a socio-economic gradient in these findings, with those of greatest advantage least bothered by poor appetite or overeating, although following adjustment for other factors only the two extreme quintiles of socio-economic area status remained significantly different. The ability of economic advantage to buffer against any personal economic stress arising from the pandemic lockdown (e.g. losing or being stood down from employment, business downturn) may be one explanation. Greater access to health and well-being services and preventive healthcare may be another.

These findings of adult eating behaviour concerns in Australia during the early stages of the COVID-19 pandemic do bear some similarities to what has been observed in other countries. The proportion of those who reported being bothered by poor appetite or overeating in this study was similar to self-reported dietary behaviours in Italy^(10)^ and Poland^(12)^. In the survey of 1932 Italians, 53 % reported that they were eating more during confinement; 42 % of survey respondents attributed their dietary change to stress, anxiety or boredom^(10)^. Another survey covering Northern, Central and Southern Italy found that 17·8 % of the sample reported reduced appetite, while 34·4 % reported increased appetite during the COVID-19 lockdown^(11)^.

### Strengths and limitations

A strength of this study is the large sample, with respondents from every state and territory of Australia, and the sample weighting to enhance national representativeness. Australia is a high-income country with a universal health and welfare system, and thus the findings of this study may not be directly applicable in other settings, including low-income countries^(32)^. Validated measures of symptoms of anxiety and depression were employed, and the survey included a question which captured level of concern about contracting COVID-19 as well as a question about the impact of the pandemic restrictions. It should be noted that the question about eating behaviour asked whether respondents were ‘bothered by poor appetite or overeating’, and does not enable these two eating behaviour constructs to be considered separately. It was not possible to determine the magnitude of any change in eating behaviour, not to determine whether consequences of this bothersome eating behaviour had manifested. It should also be noted that analysis of the association between depression scores and poor appetite or overeating is limited by the fact that the appetite question is a component of the score; thus, the OR for this should be considered with caution.

## Implications and conclusions

The findings of this study indicate that bothersome poor appetite or overeating was experienced by over 50 % of adults during the first month of COVID-19 restrictions during the first wave of the pandemic. While short periods of dietary behaviour change may not significantly impact upon weight, with longer periods of lockdown, this may become of greater public health concern. Prolonged overeating increases risk of weight gain and obesity, with obesity being a well-known risk factor for chronic disease^(33)^. Weight gain may also cause psychological distress associated with unwanted changes in body shape^(25)^. During the first wave of the pandemic, research has emerged indicating that obesity is a risk factor for greater severity of COVID-19^(34)^. Weight gain might therefore heighten distress among those already concerned about contracting COVID-19. With subsequent waves of the pandemic and variability in responses to these across different countries, longitudinal studies of eating behaviour and other behavioural risk factors are needed to understand both short-term and long-term mental and physical health impacts^(35)^.

There are factors that may limit (*older age, regional dwelling, being more accustomed to spending time at home*) or increase (*female gender, anxiety, depression, being in lockdown with children*) risk of being bothered by poor appetite or overeating for adults living in a high-income country during lockdown necessitated to control a pandemic. From a public health perspective, this research can inform universal and targeted public health promotion strategies to assist people to restore healthier eating patterns during periods of society-wide confinement and disruptions to routine. It has been suggested that cognitive behavioural therapy may be one option for improving emotional regulation and assisting development of coping strategies associated with lockdown, and e-health delivery of cognitive behavioural therapy may be appropriate for those experiencing severe distress^(36)^. As more than half of adults in this large sample reported being bothered by poor appetite or overeating, population-wide public health strategies may also be warranted for subsequent pandemic waves and periods of lockdown.
